# Chitosan-covered liposomes as a promising drug transporter: nanoscale investigations

**DOI:** 10.1039/d0ra08305d

**Published:** 2021-01-05

**Authors:** Lemaalem Mohammed, Hadrioui Nourddine, El Fassi Saad, Derouiche Abdelali, Ridouane Hamid

**Affiliations:** Laboratoire de Physique des Polymères et Phénomènes Critiques Sciences, Faculty Ben M'Sik, Hassan II University P.O. Box 7955 Casablanca Morocco mohammedlemaalem@gmail.com

## Abstract

Liposomes are small artificial vesicles spherical shaped of 50–1000 nm in diameter. They are created from natural non-toxic phospholipids membranes. Externally, they are decorated with biocompatible polymers. Chitosan, a natural polymer, demonstrates exceptional advantages in drug delivery, in particular, as liposome cover. In this paper, Molecular Dynamics simulations (MD) are performed in the coupled NPT-NPH and NVT-NVE statistical ensembles to study the static and dynamic properties of DPPC membrane-bilayer with grafted cationic chitosan chains, with added Cl^−^ anions to neutralize the environment, using the Martini coarse-grained force-field. From the NPT-NPH MD simulations we found a chitosan layer *L*_DM_ ranging from 3.2 to 6.6 nm for graft chains of a degree of polymerization *n*_p_ = 45 and different grafting molar fractions *X*_p_ = 0.005, *X*_p_ = 0.014 and *X*_p_ = 0.1. Also, the chitosan chains showed three essential grafting regimes: mushroom, critic, and brush depending on *X*_p_. The DPPC bilayer thickness *D*_B_ and the area per lipid *A*_l_ increased proportionally to *X*_p_. From the NVT-NVE MD simulations, the analysis of the radial distribution function showed that the increase of *X*_p_ gives a more close-packed and rigid liposome. The analysis of the mean square displacement revealed that the diffusion of lipids is anomalous. In contrast, the diffusion of chitosan chains showed a normal diffusion, just after 100 ps. The diffusion regime of ions is found to be normal and independent of time. For the three identified regimes, the chitosan showed a tendency to adhere to the membrane surface and therefore affect the properties of the liposomal membrane.

## Abstract 2

In this paper, we studied the graft chitosan conformation and its influence on the liposome membrane structure and dynamics as a function of the grafting molar-fraction.

## Graphical abstract

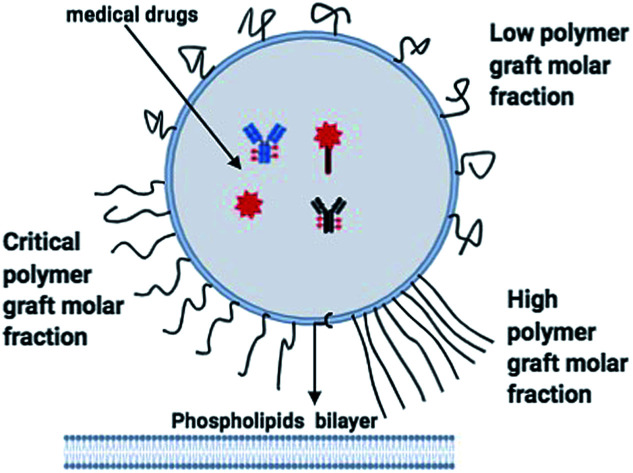

## 1 Introduction

Recently, liposomes have aroused scientific interest in many disciplines encompassing theoretical physics, biophysical chemistry, and colloidal science. They demonstrate an excellent ability to increase the localization of drugs in diseased cells.^1^ Thus, liposomes represent promising systems for drug delivery to target the drug to the site of action or improve the circulation time of the drug *in vivo*, aiming at side effect reduction as well as the optimization of treatments.^2,3^ Also, liposomes have been known for their huge potential as drug carriers for a variety of compounds like low molecular weight drugs, therapeutic proteins and peptides, vaccines and diagnostic agents, toxins, enzymes, antigens and antibodies, and nucleotides.^4–13^ The liposomes have been introduced in the market as efficient spherical nanovesicles,^6–14^ they are formed by a self-closed phospholipidic bilayer carrying the drug in its aqueous core suspended in water. During the formation of liposomes, the active drugs are merged either in the aqueous solution or in the phospholipids themselves. Thus, liposomes allowing the delivery of hydrophobic and hydrophilic agents. *In vivo* pharmacokinetics and bio-distribution can be controlled by the size and lipid composition of the liposomes. When using liposomes as drug carriers, they can release the contented drugs into the cytoplasm of cells that have internalized them by endocytosis. Therefore, they promote the intracellular penetration of active products. The liposomes surfaces are made by a lipid bilayer. Thus, the lipids nature can increase the contact time at the epithelial surface. For instance, transcorneal drug transport is limited by the intrinsic permeation characteristics of the corneal epithelium.^15^

Externally, liposomes are covered with polymers, which making them stable in the intravenous environment. Grafted polymers prolong the blood circulation time of the liposomes.^16–18^ Thus, the accumulation of drugs is significantly improved within diseased sites. From the literature, liposomes coated with chitosan have been used as a mucoadhesive delivery system because their positively charged surface promotes adhesion to negatively charged cell membranes.^19–21^ Besides, the adhesive ability plays a vital role in drug delivery. Mainly for prolonging retention in the gastrointestinal tract and promoting penetration into the mucus layer.^22^

Nowadays, chitosan, a natural polymer that receives increasing attention as a new material,^23^ may represent an alternative to neutral polymers, such as polyethylene glycol PEG and polyethylene oxide PEO,^6,7^ for liposomes covering. Chitosan does not only possess advantageous non-toxicity, low allergenicity, biocompatibility and biodegradability^23–26^ but also contains free charges in low pH water solution allowing interactions with the polar heads of the phospholipidic bilayers in the liposomes.^27^ Regarding the applicability of liposomes containing chitosan, several studies have highlighted the use of chitosan as an absorption-enhancing agent and have mentioned his inherent properties of mucoadhesion and penetration.^28–30^ Moreover, because of its bioadhesive properties, chitosan has also been worth in novel bioadhesive drug delivery systems for the treatment of wounds and burns as well as for local administration of drugs in treatments of diseases in the eyes, nasal, mucosa, or tumors.^31–34^ Besides, chitosan is reported to have other biological and biomedical properties, such as antitumor,^35^ antimicrobial,^36^ and antioxidant activities.^37^ Chitosan has been used in drug delivery systems in different forms, like tablets, microspheres, micelles, vaccines, nucleic acids, hydrogels, nanoparticles, and conjugates. Chitosan can be used in drug delivery systems in both implantable as well as injectable forms through oral, nasal and ocular routes. Besides, it facilitates transmucosal absorption which is important in nasal and oral delivery of some polar drugs like peptides along with protein vaccines for their administration.^38^ It is commonly used as an excipient in tablet formulation for oral medication. Chitosan microspheres have been extensively investigated for the controlled release of drugs and vaccines through oral and nasal delivery. They were prepared by complexation between the cationic chitosan and anionic compounds. For example, tripolyphosphate or alginates.^39,40^ The chitosan hydrogels are three-dimensionally structured hydrophilic polyelectrolyte which can absorb and hold up to thousands of times more fluids than their dry weights and use in drug delivery.^41^ Thus, the drugs can be loaded in the hydrogels by diffusion, entrapment, and tethering. After injection of the loaded hydrogel into the body, the drug diffuse into the other tissues.^42^ The recent development of *in situ* forming depots using chitosan-based hydrogel has attracted much attention as a new method for controlled drug release.^43^

Chitosan-coated liposomes can be investigated using different tools. In this context, Ketzasmin *et al.* reported a Dissipative Particle Dynamics (DPD) mesoscopic simulations of nanoliposomes. The nanoliposomes are constituted by lecithin and coated with a shell of chitosan.^44^ The DPD simulation showed that chitosan is deposited well on the surface of the nanoliposome, as has been reported in some experimental works.^45,46^ Mengoni *et al.* performed a dynamic light scattering with non-invasive backscattering (DLS-NIBS) experiments on a chitosan-coated liposomes system. Their results confirm the presence of the chitosan coating, which is remarked in the increase of the average size of liposomes and in the inversion of the zeta potential from negative value, for the uncoated liposomes, to a positive value for the coated ones. In addition, their results show that chitosan-coating improves the stability of liposomes. Indeed, in contrast to uncoated microemulsions,^47^ uncoated liposomes are thermodynamically unstable. Thus, they are rarely used without coating in drug delivery since they aggregate easily during storage, degradation, and delivery of drugs upon administration.^42^ In another work, Mady and Darwish investigated the chitosan-coated DPPC liposomes physicochemical properties, using drug release rate, transmission electron microscopy (TEM), zeta potential and turbidity measurement. The electron microscopy and the zeta potential confirmed the coating of DPPC liposomes with a chitosan layer. In addition, the authors concluded that chitosan increases liposome stability during drug release.^48^ M. Hasan *et al.* applied various techniques to study the interactions between phospholipid, chitosan, and curcumin showing that liposome coating has interesting advantages.^49^ After liposome coating, a flow behavior change from Newtonian to shear-thinning is demonstrated. The hysteresis loop area decreased significantly and therefore liposomal dispersion stability increased significantly with the addition of the chitosan layer. Also, improvement of mechanical stability is demonstrated. In addition, the liposome membrane structure was not affected by the chitosan layer.

**Fig. 1:**
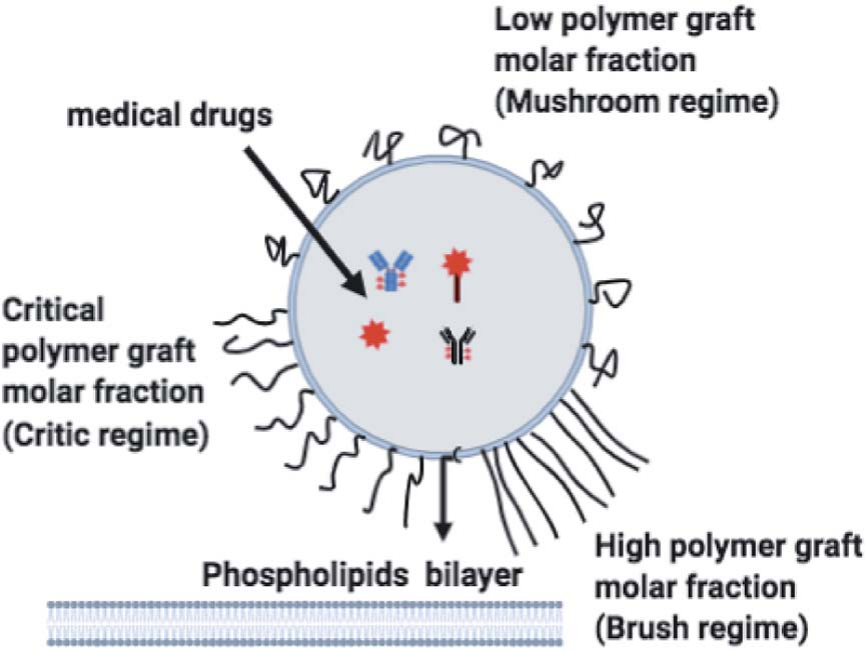
Schematic representation of liposomes covered with polymers: the polymers form a shield that protects the liposomes and play a lubrification role in the intravenous environment. The polymers can show different conformation depending on the grafting molar fraction.

**Fig. 2:**
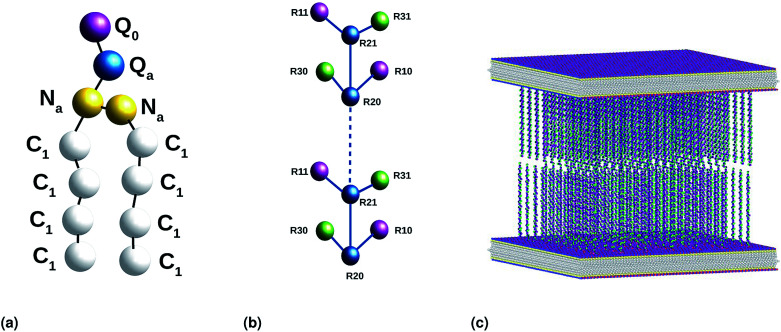
Coarse-grained representation of the simulated system: (a) DPPC molecule, (b) chitosan chain and (c) the initial distribution, two bilayer membranes opposite to each other with grafted chitosan chains. This initial configuration was generated using the Moltemplate package (https://moltemplate.org/), and visualized using the Ovito vizualisation tool (https://ovito.org).

The chitosan has interesting applications in drug delivery. Thus, it is necessary to better understand the microscopic properties of this promising polyelectrolyte and their dependence on the grafting molar fraction. As schematically presented in the Fig. 1, chitosan can be found in different conformation: mushroom, critic or brush as observed for neutral polymers.^50^ Thus, Molecular Dynamics simulations (MD) have a great advantage in the analysis of polymer conformation. Simultaneously, molecular dynamics is a powerful tool for studying the interaction between particles under different microscopic conditions. The liposome diameter range is 50–1000 nm. To mimic two adjacent liposomes the simulated system consists of two opposed lipid bilayers, where the opposed monolayers containing incorporated DPPC-chitosan lipo-polyelectrolyte. The confined area between the bilayers is filled with water molecules and Cl^−^ counterions. This system is supposed to mimic portions of two adjacent liposomes. A schematic representation is presented in Fig. 2.

In this paper, we present and analyze the results obtained from NPT-NPH and NVT-NVE molecular dynamics simulations. The structure and dynamics of the system components are found depending on the grafting molar fraction. The chitosan layer increases and the chitosan dynamics decrease proportionally to the grafting molar fraction *X*_p_. As interesting results, the DPPC area per lipid and the DPPC thickness increase with increasing *X*_p_. In contrast, the DPPC dynamics showed a slight decrease with increasing *X*_p_. Thus, chitosan incorporation can affect slightly liposome properties. Chitosan chains and ions are adhered to the liposome surface. All these features indicate that chitosan can be used as a promising covering for liposomes designed for drug delivery.

## 2 Simulation method

### 2.1 Interaction potentials

We used the Martini force field that reproduces the free partitioning energies between the polar and nonpolar phases of a large number of chemical compounds, to describe the interaction potentials of DPPC lipids, water, anions and chitosan.^51–54^ In the Martini coarse-grained model, approximately four heavy atoms have been grouped into a single coarse-grain bead, whose chitosan repetitive unit is composed of three sub-monomers (R1–R2–R3). Such units are bonded repetitively to construct chitosan chains with the desired polymerization degree. As described in detail in the original work,^54^ the R1 bead comprises atoms of the hydroxyl side chain, the bead R2 comprises most of the atoms of the polymer backbone and the bead R3 maps the hydroxyl and amino groups opposite to the side chain R1. The three beads have different hydrophilicity, and the R3 bead can become protonated and is then well presented as R3^+^. The bonded interactions are parameterized by fitting the distributions of corresponding bonds, angles and dihedrals to the all-atom MD simulation results. Also, the coarse-grained bond and angle distributions match well with corresponding distributions in all-atom MD simulation. The non-bonded interactions are parameterized using the MARTINI CG force field of carbohydrates.

In the coarse-grained model, all particle pairs *i* and *j* at distance *r* interact *via* Lennard-Jones *V*_LJ_ and coulombic *V*_C_ interactions:1
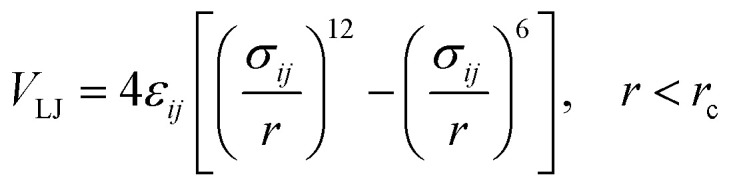
with *ε*_*ij*_ the potential depth, and *σ*_*ij*_ the bead radius.2
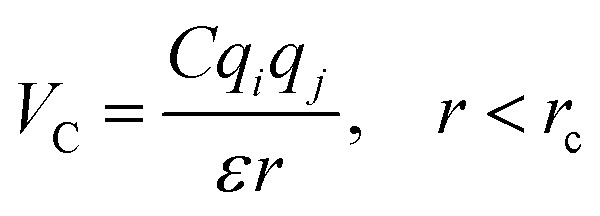
with, 
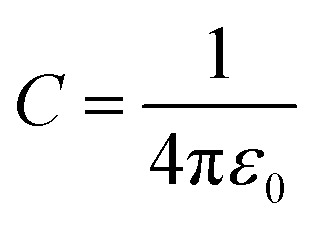
, where *ε*_0_ the vacuum permittivity, and *ε* = 15 the medium dielectric-constant. For the DPPC lipids and chitosan chains, the bond interactions between adjacent pairs of beads are described by a harmonic potential *V*_bond_(*r*), and a cosine squared harmonic potential *V*_angle1_(*θ*) is used for angle interactions between triplets of DPPC beads:

3 *V*_bond_ = *K*_b_(*r* − *r*_0_)^2^

4 *V*_angle1_ = *K*_a_(cos(*θ*) − cos(*θ*_0_))^2^

For the chitosan polyelectrolyte, the angle interaction is computed using a harmonic potential *V*_angle2_. Taking account the chitosan chains flexibility, a Fourier dihedral interaction potential *V*_dihedral_ is used to compute the interaction between quadruplets of bonded beads.

5 *V*_angle2_ = *K*_a2_(*θ* − *θ*_0_)^2^

6
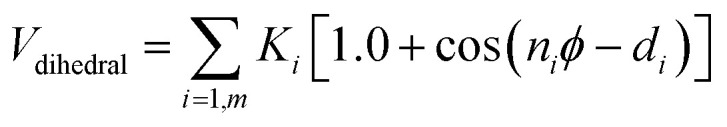


The parameters for bonded and nonbonded interactions were taken from the recently available study for DPPC, anions, and water from ref. 51–53, and for the chitosan polyelectrolyte from ref. 54. The nonbonded interaction between the chitosan polyelectrolyte monomers, water beads, Cl^−^ anions, and DPPC beads are calculated using the Lorentz–Berthelot mixing rules,^55,56^ where 
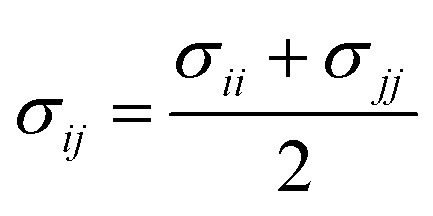
 and 
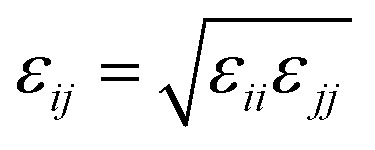
.

### 2.2 Simulation details

The simulated system consists of two membranes bilayer, with a grafted chitosan model, where each monolayer consists of 4624 DPPC lipid molecules with an area per lipid *A*_l_ = 0.49 nm^2^. Different grafting molar fractions are tested (0.005, 0.014, and 0.1), with a degree of polymerization *n*_p_ = 45. These parameters are used in previous experimental and simulation works for the neutral polymer polyethylene glycol (PEG).^50^ Thus, if chitosan has the same physical properties as PEG in term of the length of the polymer layer and the effect on the liposome membrane, it will be preferable that it be used as an alternative to PEG to cover liposomes for drug delivery applications because of its pharmaceutical advantages.

The confined area between the membranes bilayer is hydrated by 100 000 explicit water beads. The neutral charge was realized by adding Cl^−^ anions to compensate the positive charge of chitosan sub-monomer R3^+^. Molecular dynamics simulations were performed using the LAMMPS software package.^57^ Initially, the two DPPC membrane-bilayers with incorporated DPPC-chitosan lipopolyelectrolyte, opposed to each other, are generated in a cubic simulation box of lengths (*L*_*x*_ = *L*_*y*_ = *L*_*z*_ = 50 nm). Then the confined volume between the two membrane-bilayers is filt with water and Cl^−^ ions. This preformed initial configuration was optimized using an energy minimization algorithm, by iteratively adjusting particle coordinates, for a sufficiently long time (10^6^ time-steps), a finite unitless stopping tolerance for energy (*E*_stop_ = 10^−7^) that represents the energy change between successive iterations divided by the energy magnitude, and a stopping tolerance for force (*F*_stop_ = 10^−8^ kcal Å^−1^. The iterations are stopped when the energy change between outer iterations is less than *E*_stop_, or the length of the global force vector is less than the *F*_stop_. The stopping time, at which the equilibrium is achieved, is found of the order of (10^5^) time-steps for all simulated cases. Thus, the pre-formed initial distribution of the particles in the simulation box is corrected to be in local potential energy minimum. By using this minimization algorithm, the divergence is avoided and the thermodynamics equilibrium, in the production ensembles NPT-NPH and NVT-NVE, is reached quickly.

Then, molecular dynamics simulations are performed in NPT-NPH and NVT-NVE ensembles. Where *N* is the number of coarse-grained beads, *V* is the volume of the simulated system, *T* is the temperature, *H* is the enthalpy, *E* is the internal energy and *P* is the external pressure. In the NPT-NPH simulation, which updates the position and velocity of the system particles and allows the change in volume using time integration on Nosé–Hoover style non-Hamiltonian equations of motion of the system particles of molar mass *m*_*i*_ in Dalton, without energy divergence. We calculated the equilibrium distance between the two membranes, which correspond to the length of the chitosan brush bilayer, and the thicknesses of the membranes, for different molar fractions of lipopolyelectrolyte at ambient conditions of temperature and pressure (*T* = 300 K, *P* = 1 bar), by analyzing the density profiles. For all simulations, periodic boundary conditions are used.^58^ The proposed initial distribution of the simulated system (Fig. 2) was equilibrated under the NPT-NPH conditions, by coupling the NPH barostat to the Langevin thermostat,^57,59^ with the equilibrium temperature, which is proportional the particles velocities, *T* = 300 K and the damping parameter (damp = 100). This simulation updates the positions and the velocities of the particles for each time step. Also, it modifies the volume to reach the imposed equilibrium conditions on the external pressure and the temperature. From 10^5^ to 10^6^ time-step (*δt* = 10 fs), the area between the opposed membranes bilayer remains practically constant and then the layer of the polymer brush, along the *z*-direction, is calculated from the density profiles.

The second simulation is a Brownian dynamics (BD) performed on the NVT-NVE ensemble by coupling the Langevin thermostat which models an interaction with an implicit background solvent with the equilibrium temperature, which is proportional the particles velocities, *T* = 300 K and the damping parameter (damp = 100), to the NVE integration scheme which performs a constant NVE simulation to update the position and the velocity of coarse-grained beads.^60^ In this simulation, we investigate the micro-distribution (through the radial distribution function *g*(*r*)) and dynamics (through the mean square displacement and the diffusion coefficients) of DPPC lipids, chitosan molecules, and Cl^−^ anions. The simulations were run for 10^6^ time-step, with a time step *δt* = 10 fs.

**Fig. 3:**
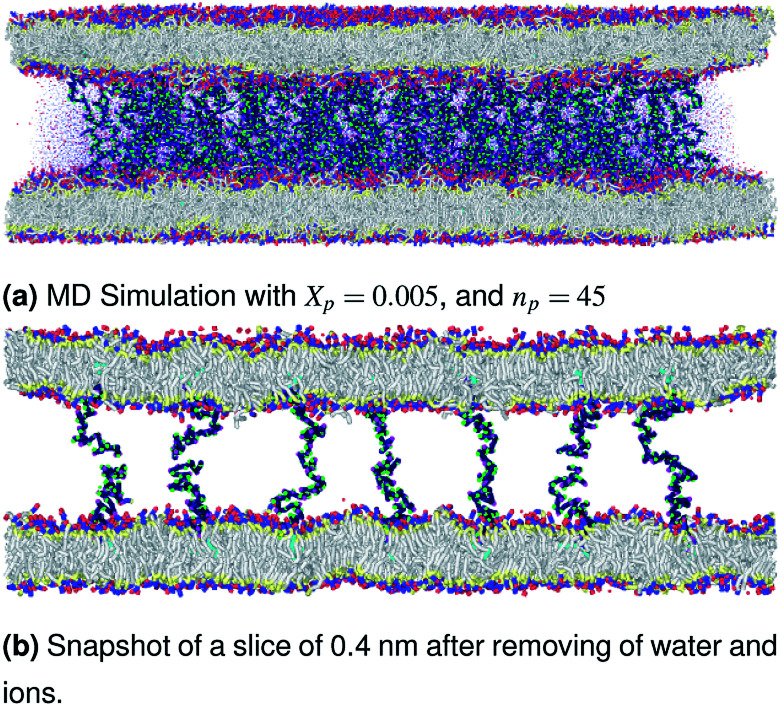
Snapshot of the NPT-NPH simulation at *T* = 300 K and *P* = 1 atm of the polyelectrolyte conformation in the mushroom regime using the Ovito visualization tool.^61^ The model membranes composed of DPPC lipid bilayer are decorated with a molar fraction *X*_p_ = 0.005 of DPPC-chitosan lipopolyelectrolyte with the same degree of polymerization *n*_p_ = 45. The whole is hydrated by water molecules. After one nanosecond (10^5^) time-step *δt* = 10 fs, the simulated system is equilibrated and the membrane–membrane distance remains constant. The chains do not interact and behave almost like isolated grafted chains.

**Fig. 4:**
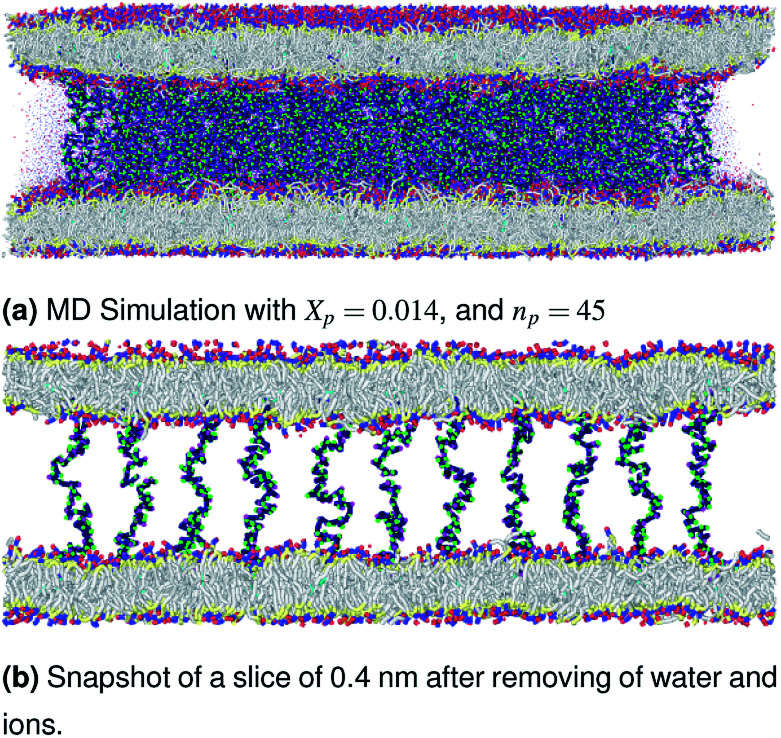
Snapshot of the NPT-NPH simulation at *T* = 300 K and *P* = 1 atm of the polyelectrolyte conformation in the critic regime. The model membranes composed of DPPC lipid bilayer are decorated with a molar fraction *X*_p_ = 0.014 of DPPC-chitosan lipopolyelectrolyte with the same degree of polymerization *n*_p_ = 45 and the whole is hydrated by water molecules. The transition between mushroom and brush regimes take place when the chitosan chains begin to overlap.

**Fig. 5:**
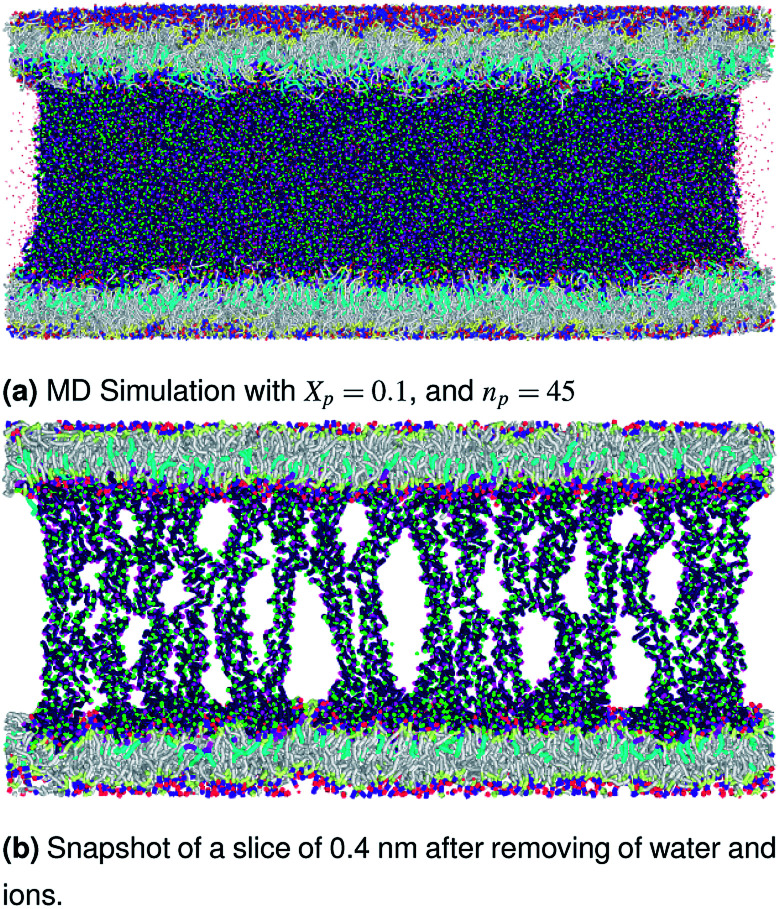
Snapshot of the NPT-NPH simulation at *T* = 300 K and *P* = 1 atm of polyelectrolyte conformation in the brush regime. The model membranes composed of DPPC lipid bilayer are decorated with a molar fraction *X*_p_ = 0.1 of DPPC-chitosan lipopolyelectrolyte with the same degree of polymerization *n*_p_ = 45 and the whole is hydrated by water molecules. The chitosan chains are extended from the grafting surface forming a brush-like conformation.

The visualizations of the NPT-NPH simulations, the snapshots shown in Fig. 3–5 correspond to the final step are also the most probable conformations, as we notice a thermodynamics equilibrium for all simulated cases after 10^5^ time-step, *i.e.*, one nanosecond.

More pieces of information on the energy minimization algorithm and the different statistical ensembles, used in this work, are available in detail on the online version of the LAMMPS package (http://lammps.sandia.gov). All interaction potentials parameters, needed for the MD simulation, are listed in detail in Tables 1–4. More detailed information about the MARTINI coarse-grained representation and parametrization of the coarse-grained interaction potentials of lipids, polymers (monomers), water and ions, can be found on the MARTINI home page (http://cgmartini.nl/).

**Table:** Non-bonded interaction potential parameters

Pair type	*σ* (Å)	*ε* (kcal mol^−1^)
Q_0_–Q_0_, C_1_–C_1_	4.7	0.836521
Q_a_–Q_a_, MW–MW	4.7	1.195030
N_a_–N_a_, Q_0_–N_a_, Q_a_–N_a_	4.7	0.956024
Q_0_–Q_a_	4.7	1.075527
Q_0_–C_1_, Q_a_–C_1_	6.2	0.478012
MW–C_1_	4.7	0.478012
N_a_–C_1_	4.7	0.645316
Q_0_–MW, Q_a_–MW	4.7	1.338434
N_a_–MW	4.7	0.956024
R1–R1, R2–R2, R1–R2	4.7	1.195030
R1–R3^+^, R2–R3^+^, R3^+^–R3^+^	4.7	1.338434
R1–MW, R2–MW, R1–Q_a_, R2–Q_a_	4.7	1.195030
R1–C_1_, R2–C_1_, R1–Q_0_, R2–Q_0_	4.7	0.999834
R1–N_a_, R2–N_a_	4.7	1.068867
R3^+^–MW, R3^+^–Q_a_	4.7	1.264701
R3^+^–C_1_, R3^+^–Q_0_	4.7	1.058124
R3^+^–N_a_	4.7	1.131183
Cl^−^–Cl^−^	4.83	0.012785
Cl^−^–C_1_, Cl^−^–Q_0_	4.765	0.103416
Cl^−^–MW, Cl^−^–Q_a_	4.765	0.123606
Cl^−^–N_a_	4.765	0.110556
Cl^−^–R2, Cl^−^–R2	4.765	0.123606
Cl^−^–R3^+^	4.765	0.130812

**Table:** Bonded interaction potential parameters

Bond type	*r*_0_ (Å)	*K*_b_ (kcal mol^−1^)
Q_0_–Q_a_	4.7	2.98
Q_a_–N_a_	4.7	2.98
N_a_–N_a_	3.7	2.98
N_a_–C_1_	4.7	2.98
C_1_–C_1_	4.7	2.98
R2–R2	5.212	7.17
R2–R3	1.934	7.17
R1–R1	2.494	7.17

Angle interaction potential parameters

**Table:** 

Angle type	*θ*_0_ (°)	*K*_a1_ (kcal mol^−1^)
Q_a_–N_a_–N_a_	120	5.97
N_a_–C_1_–C_1_	180	5.97
C_1_–C_1_–C_1_	180	5.97

**Table:** 

Angle type	*θ*_0_ (°)	*K*_a2_ (kcal mol^−1^)
R10–R20–R30	123.76	107.553
R20–R21–R22	163.78	83.652
R20–R21–R11	70.94	107.553
R10–R20–R21	90.88	23.9006
R30–R20–R21	135.94	71.7017
R31–R21–R20	58.01	191.205

**Table:** Dihedral interaction potential parameters

Dihedral type	*ϕ*_0_ (°)	*K*_i_ (kcal mol^−1^)	*n*_i_
R10–BR0–R21–R11	175.62	1.91205	3
R10–R20–R21–R31	−28.59	2.39006	3
R30–R20–R21–R11	36.57	4.3021	3

## 3 Results and discussions

### 3.1 Length of the chitosan layer

Chitosan chains have *n*_p_ Kuhn segments and carry *n*_p_ elementary charges *e*, where the so-called Kuhn segment is composed of three attached beads (R1–R2–R3^+^). For each chain, the same number *n*_p_ of mobile monovalent Cl^−^ counterions distributed in the solution above the membranes. In a recent report,^50^ we studied the grafted PEG conformations using MD simulations. We showed that, when the lipo-polymers are incorporated into a bilayer membrane, the PEG neutral polymers exhibit three conformations, namely mushroom, critic and brush regimes, for a fixed degree of polymerization *n*_p_ = 45. These conformations are found depending on the lipo-polymer grafting molar fraction (*X*_p_). Thus, the chitosan chains are expected to show the same conformations.

#### 3.1.1 Length of the chitosan layer for low grafting molar-fraction

At high salt concentration and low grafting molar fraction, the conformation of chitosan chains is independent of the grafting molar fraction and salt concentration, and each chain is isolated from its neighbors. Therefore, there are no collective stretching effects. For simplicity, we consider the chitosan chains as linear homopolymer chains in a homogeneous environment and disregard all monomer–monomer interactions, including the hard-core ones that prevent the chains from overlapping and the coulombic interaction between charged monomers. For high molecular weights, all such polymers fall into the same universality class of non-avoiding random walks. Thus, they exhibit a degree of common behavior. For instance, their length scales with their molecular weight with a universal exponent, *ν* = 1/2. This behavior is common for natural and artificial polymers. For maximum simplicity, we select the freely-jointed chain, where each monomer has a fixed length *a*, and the joint between sequential monomers is completely flexible. In this regime, the polymer chains have a random distribution.7
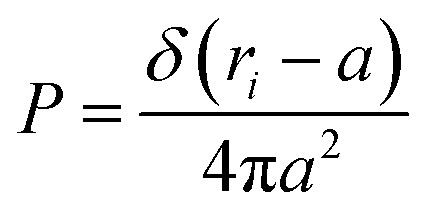
where *r*_*i*_ the modulus of the vector **r**_*i*_. The factor in the denominator ensures that the distribution is properly normalized such that:8
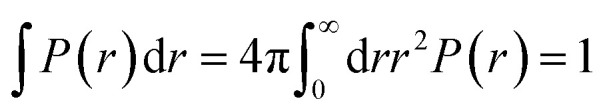


The size of the particular conformation can be characterized by the length of its end-to-end vector:9
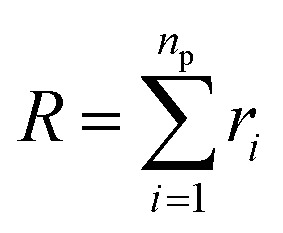
by averaging the length of this vector over all possible conformations, *R*_0_, can then be used as a measure for the length of the polymer. Since the simple average, 〈|*R*|〉, is mathematically difficult to evaluate, then it is common practice to use the alternative root-mean-square average:10
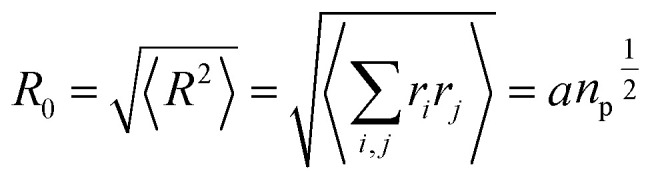


#### 3.1.2 Length of the chitosan layer for the critical grafting molar-fraction

The critic regime is an intermediate regime between the mushroom and brush regimes. In this regime, the chitosan chains are not totally stretched but the inter-chains interaction is significant. Thus, the chains begin to overlap. For chitosan chains with a fixed polymerization degree *n*_p_, the critical regime depends only on the grafting molar fraction *X*_p_. Thus, the transition mushroom-brush occurs at 
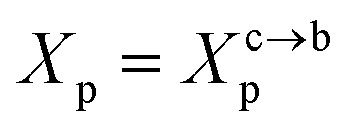
 such as:11
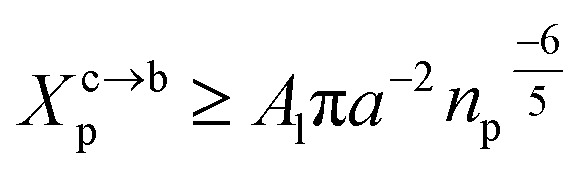
where *A*_l_ is the area of the membrane by lipids, as we will calculate in Section. 3.4, is found of the order of (*A*_l_ = 0.62 nm^2^) for the critical grafting molar fraction 
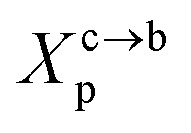
. For a graft chitosan with (*n*_p_ = 45), the transition between the mushroom and brush conformations must take place at 
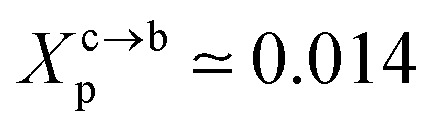
 for fluid phase membranes. The effect of the chitosan coating on the area per lipid will be discussed in detail in Section 3.4.

#### 3.1.3 Length of the chitosan layer for high grafting molar-fraction

The layer of the chitosan brush in contact with a salt solution depends on the Bjerrum length *l*_B_ defined as the distance at which two elementary charges *e* interact with thermal energy *k*_B_*T* = *e*^2^/(*εl*_B_) in a solvent with dielectric constant *ε*, and, of course as well as for neutral brush, to the molar fraction of graft polyelectrolyte *X*_p_, as presented firstly by Milner^62,63^ and proved experimentally by Kenworthy *et al.*^64^ We note that in this work we simulate a neutral system (*n*(R3^+^) = *n*(Cl^−^)), so the dependence of the polyelectrolyte layer on the salt concentration per chain is not discussed in this paper. This dependence is discussed in detail by Zhulina and Rubinstein.^65^ For very high molar fraction, 1 chains create an electric field which stretches each chain beyond the size of a free chitosan *R*_0_ (eqn (10)). The size of the chain in this regime, called the Pincus regime,^65^ is determined by the balance of the electrostatic energy per chain *F*_elec_12
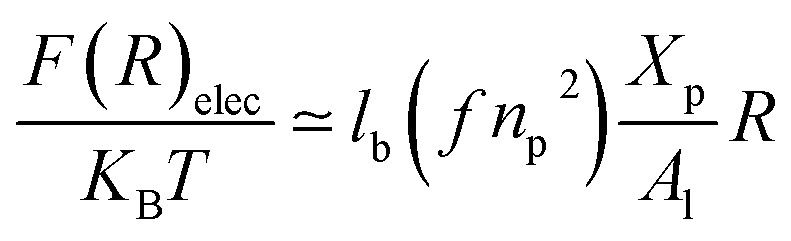
where *f* is the degree of chain ionization (fraction of charged Kuhn segments), and *F*_elast_ is the elastic free energy per chain13
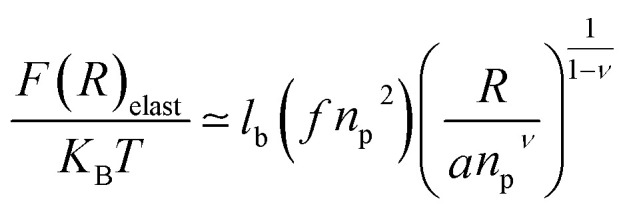
with *ν* = 3/5 in good solvent conditions, giving a brush height14
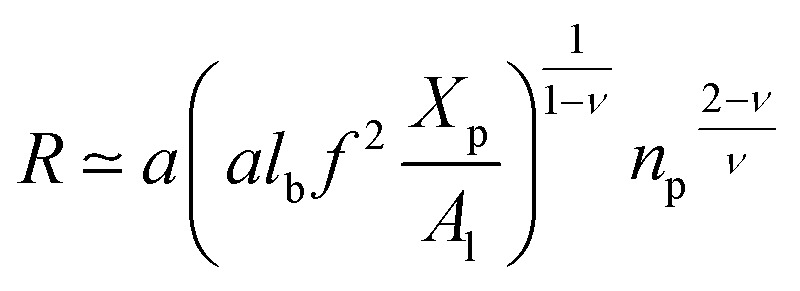


While the chitosan is highly charged (*f* = 1) with condensed Cl^−^ counterions, there is no Pincus regime and *X*^C^_p_ > *X*^osm^_p_, with *X*^osm^_p_ the osmotic molar fraction. For osmotic brushes, the grafting molar fraction is large enough that the counterions become highly localized, thus determining the properties of the brush. Therefore, in this case, the chitosan layer is determined by the balance of the osmotic pressure of the counterions and the elastic energy of the chains, which gives the following formula.^65^

15 *R* ∼ *an*_p_

On the other hand, the energy of short-range excluded volume interactions between monomers becomes higher than the counterion osmotic contribution to the brush free energy. In this case, Ekaterina and Rubinstein demonstrate that PE brushes are in a quasi-neutral brush regime and have the same properties as neutral brushes.^65^ Then the chitosan layer can be determined through the *mean field theory*. In the brush regime, the chitosan chains are extended and the problem is essentially unidimensional. The chains are confined in the normal direction to the grafting surface. In the framework of the mean-field theory, for a grafted polymer chains, the one-dimensional version of the average field expression for free energy is:^66^16
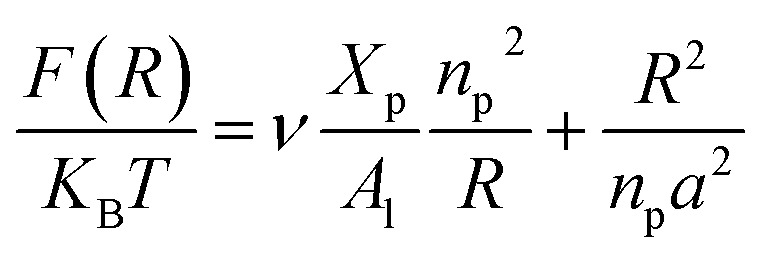
Here, for the polyelectrolyte brush, *ν* = *a*^3^ the excluded volume per repetitive unit (R1–R2–R3), *X*_p_ is the molar fraction of the graft polyelectrolyte and *A*_l_ is the area per lipid. Minimizing the free energy of eqn (16) with respect to *R* (the end-to-end distance of the chain), *i.e.*, 
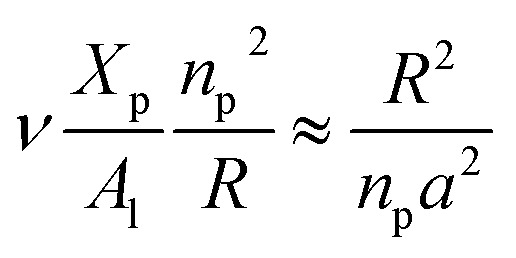
 gives the following expression for the equilibrium length of the polymer brush:17
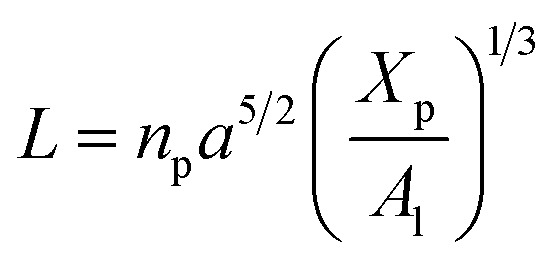


### 3.2 Chitosan monomers and Cl^−^ counterions density distribution

**Fig. 6:**
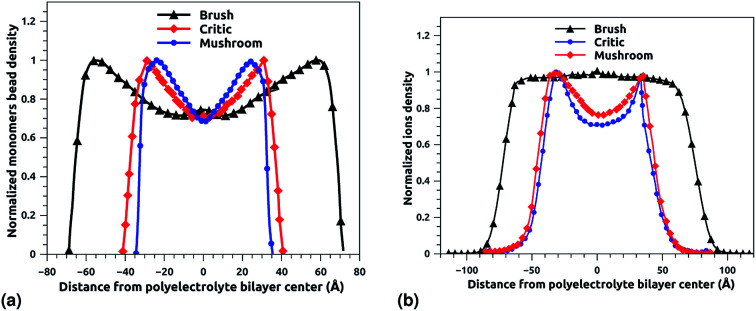
The density profile in the *z*-direction of the confined chitosan chains (a) and Cl^−^ counterions (b).

Fig. 6a and b depict the density profile of grafted chitosan chains and Cl^−^ counterions, respectively, using a degree of polymerization *n*_p_ = 45, for three different molar fraction. It has been observed that the distance between two parts of adjacent liposomes, which are supposed as flat surfaces, increases with the increase of the grafting molar fraction. For bilayer of graft chitosans, in the ambient conditions of temperature and pressure, the density profile of the chitosan monomers present as symmetric curves centered in the middle of the *z*-direction of the simulation box (*L*_*z*_ = 0), giving that there is no remarkable interpenetration between the two polyelectrolytes layers. Thus, the chitosan bilayer is considered as polyelectrolyte brush in weak interpretation regime.^66^ Then, the length of the chitosan layer can be estimated as *L*_DM_ = *d*/2. According to Fig. 6a, the monomer density decreases away from the grafting surface. Noted that, larger *X*_p_ implies a larger number of monomers. Therefore, an increase in *X*_p_ implies that we find a larger value of the monomer concentration at a given distance from the wall, regardless of the extent of lowering of the monomer concentration away from the grafting surface. Fig. 6a demonstrating the existence of finite monomer concentration near the end of the chitosan chains. Thus, chitosan adheres to biological membranes. Fig. 6b shows the counterion concentration distribution for different *X*_p_. The counterion concentration distribution almost obeys the monomer concentration distribution for the mushroom and critic regimes. In contrast, for the brush regime, the counterions are pushed away from the grafting surface by sterical effects. Therefore, the counterion concentration decreases near the end of the chains as it is observed for the monomer concentration distribution. For the brush regime, we witness a more uniform counterion density distribution, with a small increase at the end of the chitosan chains, such an increase is observed also in the monomer's density. Thus, the counterions remain tightly bound to the brushes. Obviously, there is a quantitative variation between the monomer and counterion density profile, particularly near the end of chains, at that location the monomer density decreases steeply while the corresponding counterion density shows a much flatter decrease.

In Table 5 we present the chitosan layers obtained by the present MD simulations at the NPT-NPH statistical ensemble under the ambient condition of pressure and temperature, for different molar fractions of chitosan (0.005, 0.014 and 0.1) using a lipopolyelectrolyte composed of a DPPC molecule bound to a chitosan polyelectrolyte chain of degree of polymerization *n*_p_ = 45, for these parameters the transition mushroom-brush lipopolyelectrolyte molar fraction is *X*_p_ = 0.014, from Fig. 3–5, it is clear that for *X*_p_ = 0.005 the lipopolyelectrolyte show a mushroom regime, for *X*_p_ = 0.014 show a critical regime and for *X*_p_ = 0.1 show a brush regime. From molecular dynamics simulation, we find that the thickness of the chitosan layer increases with the increase of the grafting molar fraction. The chitosan layer for *n*_p_ = 45 and *X*_p_ = 0.005, 0.014 and 0.1 are found around 3.2 nm, 3.5 nm and 6.6 nm, respectively. Previous experimental results for the PEG layer with the same parameters, using the X-ray diffraction method, give a polymer layer in the range of 3.75–6.6 nm.^64,67^ The MD results for chitosan polyelectrolyte are in good agreement with the experimental findings for PEG neutral polymer. Thus, the incorporation of DPPC-chitosan lipopolyelectrolyte prevents the adhesion liposome–liposome even with a small molar fraction of grafted polyelectrolyte, and they form a layer which protects the liposome from adhesion phenomena and from supposed system immune attack. Then, the chitosan can be used as an alternative for liposome covering.

**Table:** Thickness of the polyelectrolyte layer obtained by MD simulations *L*_DM_ for DPPC–chitosan_*n*_p__ lipopolyelectrolyte grafted in DPPC bilayer membranes, and from theoretical prediction *L*_Th_

*X*_p_	*n*_p_	Regime	*L*_DM_ (nm)	*L*_Th_ (nm)
0.005	45	Mushroom	3.2 ± 0.2	3.15^a^
0.014	45	Critic	3.5 ± 0.2	3.47^b^
0.1	45	Brush	6.6 ± 0.2	6.68^b^

a Polymer layer from eqn (10), with *a* = 0.47 nm.

b Polymer layer from eqn (17), with *a* = 0.47 nm and *A*_l_ = 0.7 nm^2^.

### 3.3 DPPC bead density distribution

**Fig. 7:**
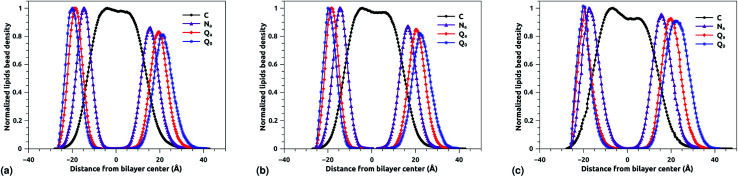
Reduced density profile of DPPC beads: (a) reduced density in the mushroom regime, (b) reduced density in the critic regime, (c) reduced density in brush regime.

The bead density profile along the normal of the membranes-bilayer is analyzed to investigate the effect of chitosan grafting density on the DPPC bilayer-membrane thickness *D*_B_ and the hydrocarbon thickness *D*_HH_. The *D*_B_ and *D*_HH_ were calculated for the three grafting regimes, at the same conditions of external-pressure and temperature (*T* = 300 K and *P* = 1 bar). Fig. 7a, b and c represent the density profiles of all bead types (C, N_a_, Q_a_, and Q_0_) for the mushroom, critic, and brush regimes, respectively. The beads density profiles across the membrane show a slight extent and a slight decrease in the monolayer in which the lipo-polyelectrolyte is incorporated for comparison to the liposome intra-monolayer. This deviation increase proportionally to the grafting molar fraction *X*_p_. Previous MD simulation study on DPPC bilayer-membranes, without polymer coating, revealed *D*_B_ = 3.85 nm.^68^

In the present study, the *D*_B_, given by the distance between the maxima of the density profile of Q0 beads, amounts to 4.1, 4.16 and 4.2 nm for mushroom, critic, and brush regime, respectively. Thus, the incorporation of lipo-polyelectrolyte increases the membrane thickness from 0.15 to 0.35 nm depending on *X*_p_. For the hydrocarbon, the bilayer beads distribution is a Gaussian like, since the two density peaks have overlapping, so they are unresolvable and they look like one peak. A closely related quantity is the half-width at half maximum (FWHM). Thus, the FWHM is used to define hydrocarbon thickness. According to this concept, the hydrocarbon thickness is found 2.75 nm, 2.79 nm and 3.05 nm for mushroom, critic and brush regimes, respectively. From the literature, Norbert *et al.* have performed an experimental study combining the analysis of X-ray and neutron ULV data to measure DPPC bilayer *D*_B_ and DPPC hydrocarbon *D*_HH_ thicknesses.^69^ They found *D*_B_ around 3.9 nm and *D*_HH_ around 2.85 nm. In comparison to their works, our results are in good agreement with the experimental findings, in terms of *D*_B_ and *D*_HH_.

### 3.4 Area per DPPC-lipid in DPPC-liposome with incorporated DPPC-chitosan lipo-polyelectrolyte

The simulations were performed at the ambient conditions of temperature and pressure (*T* = 300 K, *P* = 1 bar). For these conditions, the membrane bilayer is found in the lamellar phase giving an area per lipid *A*_l_ around 0.6 nm^2^, 0.62 nm^2^, and 0.66 nm^2^ for mushroom, critic, and brush regimes, respectively. This change of the area per lipid is due to the steric interactions between the chitosan chains, which are collected in the brush regime. Thus, giving rise to the lateral pressure which will tend to stretch the lipid membrane. Among other things, this will change the membrane permeability properties, by chain fusion. Experimental proof of such lateral expansion, is provided by the increase of the movement freedom of lipid chains with the incorporation of lipopolymer into bilayer membranes.^70^

The extent of the resulting membrane expansion is determined by Marsh^71,72^ using a precise theoretical treatment using a virial equation of state as:18
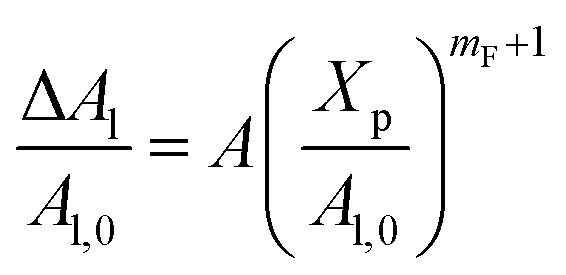
with, 
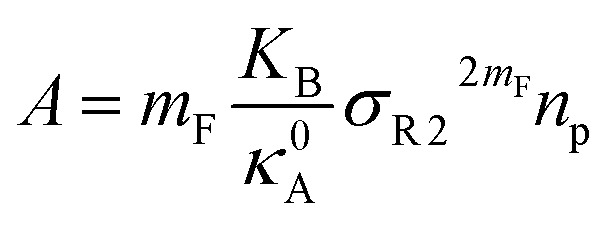
, such as, *A*_l_ the area per lipid in the presence of grafted chitosan brush, *A*_l,0_ the area per DPPC lipid in the absence of the chitosan brush, *X*_p_ the grafting molar fraction and *m*_F_ a critical exponent, which can take two values according to the used theoretical approach, 
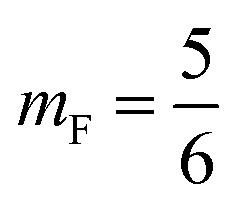
 for the scaling theory or 
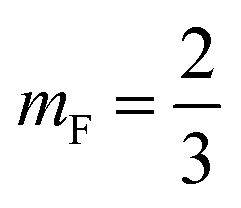
 for the mean-field theory.^71,72^ From the present MD simulation, the membrane expansion is found around 0, 0.03 and 0.1 for mushroom, critic and brush regimes, respectively. The obtained values cover the theoretical predictions (Fig. 4 of ref. 71).

### 3.5 Radial distribution function (RDF)

The radial distribution function (RDF) shows the probability of finding a particle distributed around another central particle. It provides information on the local structure of coarse-grained beads in a group defined among the various beads of the system.19
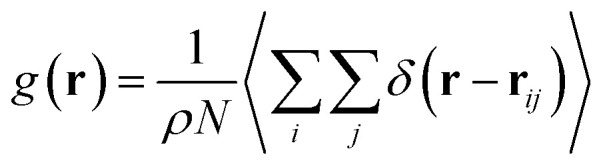
where, *ρ* = *N*/*V* is the density of the system, *N* the number of particles and *V* the volume of the simulation box.

**Fig. 8:**
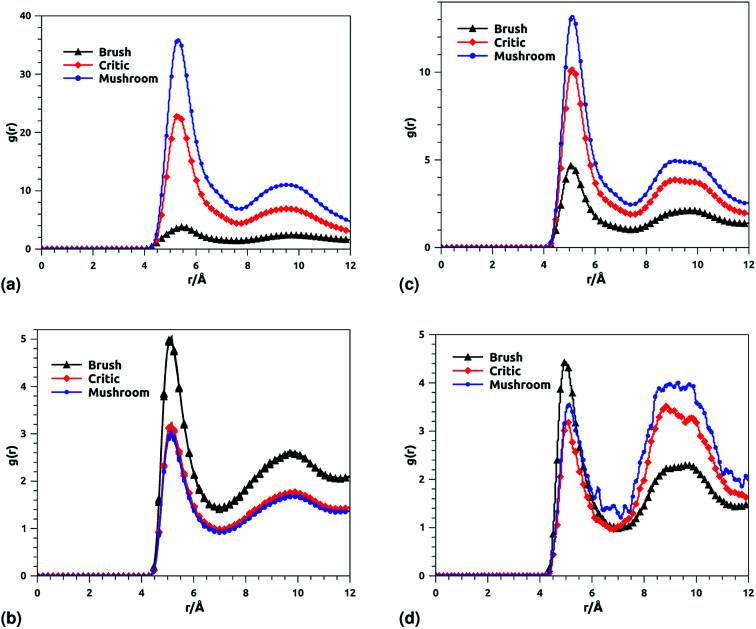
RDF of membrane grafted chitosan components: (a) RDF of the group of chitosan molecules, (b) RDF of the group of lipid molecules, (c) RDF of the group of (chitosan molecules + Ions), (d) RDF of the group of ions.

Fig. 8 shows the radial distribution function (RDF), which gives the probability of finding a coarse-grained bead at a distance *r* from another bead of the same considered group of molecules. For polyelectrolyte beads group (Fig. 8a), in the brush regime, when the chains are more stretched, the polyelectrolyte RDF has one narrow peak, at *r* = 5.2 Å, corresponding to an ordered monomers distribution, which represents the inter-chain interaction. In the mushroom regime, when the chains are congested, the polyelectrolyte RDF has two narrow peaks, the first peak, appearing at *r* = 5.2 Å, represent the intra-chains interaction and a second large peak centered at *r* = 9 Å due to the strong correlation between the chitosan monomers. Inspection of the radial distribution function confirms that, for brush, critic and mushroom regimes, the membrane bilayer is in a fluid phase, the latter is characterized by an ordered distribution at a short distance and a disordered distribution at long distance. The two main peaks, located at *r* = 5.2 Å and 9 Å, respectively, of the RDF between lipids, increase proportionally to the graft polyelectrolyte molar fraction, *i.e.*, the membrane lipids are more congested for the brush regime (Fig. 8b). The incorporation of lipopolymer affects the liposome structure positively, the stability of DPPC lipids bilayer increases with the increase of the grafting molar fraction. Fig. 8c shows the RDF of the charged group in the system (polyelectrolyte + ions), two main peaks are observed at *r* ≈ 5 Å and *r* ≈ 9 Å, respectively. The RDF main peaks are higher for the low PE incorporation, in the mushroom and critic regimes, because the ions come close to the PE, while in the brush regime the ions are pushed away because of the sterical effect. Fig. 8d shows the RDF of Cl^−^ ions, the RDF present two main peaks located at *r* ≈ 5 Å and *r* ≈ 8.5 Å, respectively. The first peak is higher for the brush regime, in contrast, the second peak is higher for the mushroom and critic regimes, in the mushroom and critic regimes the ions are distributed in the whole volume. However, in the brush regime, the ions can form confinements, near the end of the chains, because of the PE-chains steric effect.

### 3.6 Dynamics properties

#### 3.6.1 Theoretical background of diffusion

Liposomes are constructed from lipid bilayers decorated with polymers and dispersed in a solvent. The membrane of considered liposome is formed by DPPC phospholipids. The dynamic behavior of these lipid bilayer systems has been studied in-depth in terms of molecular dynamics simulations using both all-atoms and coarse-grained approaches and using different experimental tools.^73–79^ Within the framework of MD simulations, the diffusion is studied by analyzing the mean-square-displacement expressed as follows:20
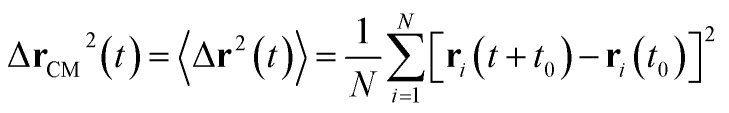


In the expression above, **r**_*i*_(*t*) represents the temporal position of a *random walker* (*i*), which can be a DPPC bead, a chitosan monomer, a water bead, or an ion. Here, *t* indicates the time and *t*_0_ is an initial time at which the *random walker* begins to move. We recall that, for the standard diffusion of simple liquid (water, ions and so on), the particles move ballistically at short times. Thus, the mean-square-displacement 〈Δ**r**^2^(*t*)〉 ∼ *t*^2^, which is followed by a crossover to Fickian diffusion, characterized by 〈Δ**r**^2^(*t*)〉 ∼ *t* for long times. On the other hand, for molecular systems such as polymers and lipids, a caging effect causes a subdiffusion regime that intermediates the ballistic and the diffusive regimes. In this case, the particles are trapped by their neighbors. Thus, this regime is characterized by 〈Δ**r**^2^(*t*)〉 ∼ *t*^*α*^, with 0 ≤ *α* ≤ 1. The value of *α* in the present of the cage effect remains astounding. In fact, there are some theoretical approaches based on some complex memories functions developed to discuss the subdiffusion laws. From the literature, Zwanzig in a series of works developed a theoretical approach based on a generalized Langevin equation (GLE),^80,81^ this approach can be adopted to discuss the subdiffusion observed in polyelectrolyte and lipids bilayer. From a mathematical viewpoint, this is simply an extension of the standard Langevin equation for simple liquid, where the friction is assumed to be determined by the instantaneous velocity of the particle. It is noted that the difficulty of understanding the subdiffusion phenomena is to mathematically explicit the cage effect that, physically speaking, depends on various physical parameters, namely, the temperature, the particle density, the particles arrangement nature and so on. In this context, the GLE is expressed as:21
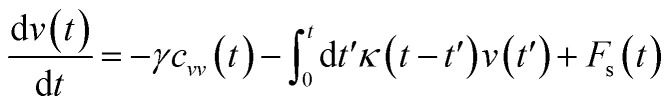
where *v* is the velocity of a moving particle, 
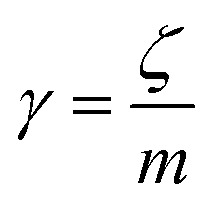
 is the relaxation rate, where *ζ* is the friction coefficient and *m* is the mass of the tracer, *κ* is the memory-function that expresses the friction retardation, and **F**_s_(*t*) is a random force felt by the moving particle due to its collisions with other particles. Thus, the random force verify the equation:

22 〈**F**_s_(*t*)·**F**_s_(0)〉 = 6*mk*_B_*T*[*γδ*(*t*) + *κ*(*t*)], *t* > 0

For this considerations VACF solves the following differential equation:23
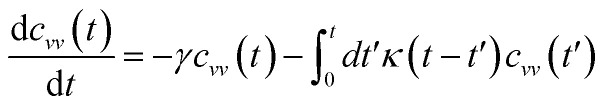


To resolve the GLE equation in the coarse-grained representation of DPPC lipids and chitosan chains, we adopt a recent memory function, proposed by Flenner,^82^ developed to study the dynamic aspect of lipid atoms. Flenner's approach is based on the Zwanzig–Mori projection method for modeling the 〈Δ**r**^2^(*t*)〉. The starting point is to consider the equation of motion for density autocorrelation function *Φ*_s_(*q*,*t*) = 〈*n*(−*q*,0)*n*(*q*,*t*)〉 of a selected particle at wave vector q,^83^24
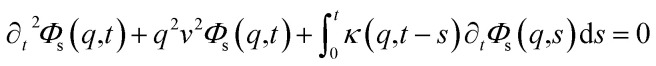
where 
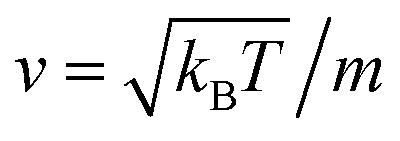
, *k*_B_ stand for the Boltzman's constant, *T* denote the temperature and *m* is the coarse-grained bead mass.

The equation of motion for the MSD in two dimension can be obtained from25
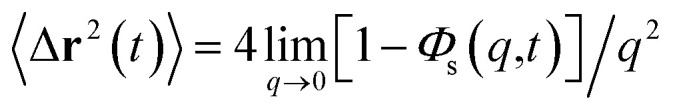
which gives26
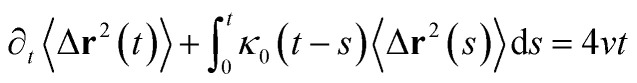
where 
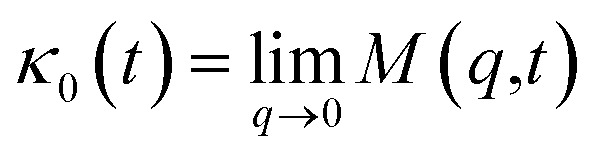
.

Flenner *et al.* propose a memory function, with explicit crossover times scales and which reproduce the three diffusion regimes, namely, ballistic, normal, and subdiffusive^82^27
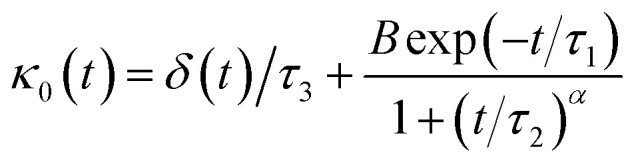
where *δ*(*t*) is the Dirac delta function, *B* is a dimensionless parameter, *τ*_1_ is the characteristic time for the crossover from the subdiffusion to the normal diffusion, *τ*_2_ is the onset time of the subdiffusion regime, and *τ*_3_ is the characteristic time for the crossover from the ballistic to the subdiffusive regime.

Analysis of this memory function revealed that, for *B* = 0, the Brownian diffusion laws, expected for ions, are recovered and the 〈Δ**r**^2^(*t*)〉 behaves like:28
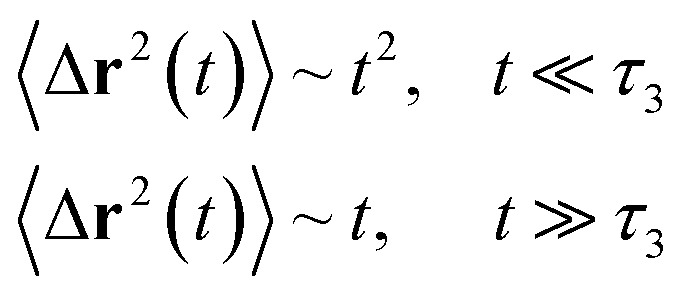


If (*t*/*τ*_2_)^*α*^ ≪ 129
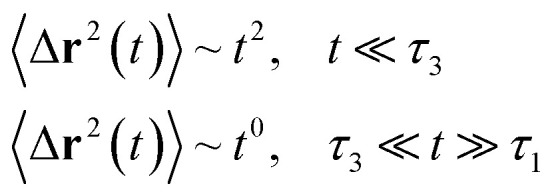


Generally, the long-term mean-square-displacement 〈Δ**r**^2^(*t*)〉 shows a normal diffusion with *α* = 1 common for all constituent particles, in good agreement with the Langevin theory. But the peculiarity of congested systems (chitosan chains and DPPC lipids) is that the mean-square-displacement exhibits a sub-diffusion regime, with 0 < *α* < 1, extended over a large time interval. This is explained by the fact that the coarse-grained beads, instead of performing a random walk, its movement is blocked by its neighbors and move with a concerted manner, in particular for DPPC lipids at *in vivo* and *in vitro* temperatures, which are generally below their melting temperature found around 314 K.

The subdiffusion phenomenon is represented mathematically in the memory function by the term of the power law. The analytical determination of the diffusion exponent *α* seems to be complex. However, a numerical computation, taking into account the term of power law in the memory function,^82^ revealed that the dynamics pass slowly from the ballistic regime to the subdiffusive regime with *α* < 1. The region of subdiffusion separating the ballistic and the normal regimes is due to the cage effect. Thus, in this region the mean-square-displacement, 〈Δ**r**^2^(*t*)〉, behaves like:

30 〈Δ**r**^2^(*t*)〉 ∼ *t*^*α*^, (*α* < 0),  *t* > *τ*_2_

The dynamics of the chitosan chains, in the mushroom and critic regimes, can be also discussed using the Rouse model,^84^ which is a simple model based on the entropic spring force acting between monomers. Thus, the equation of motion of the chitosan-beads is described by the following Langevin equations:31
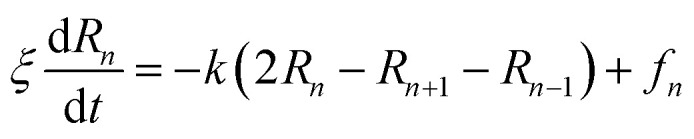
where, (*R*_1_, *R*_2_, …, *R*_*N*_ ≡ *R*_*n*_) represent the beads positions, *k* = 3*k*_B_*T*/*σ*_*R*_2__^2^ depicts the entropic spring force and *f*_*n*_ a random force representing the random collisions between monomers, *ξ* is a friction coefficient related to the diffusion coefficient, at large time, by *D*_*α*=1_ = *k*_B_*T*/*ξ*.

The solution of the Langevin equation gives the following behavior of the mean-square-displacement as a function of time.32
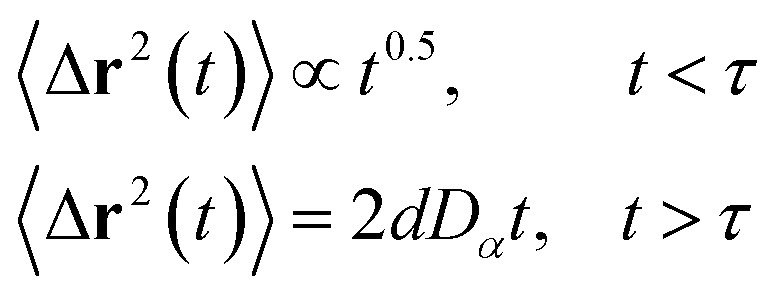
with *τ* is a crossover time, *d* the space dimension *d*, and *D*_*α*_ the normal diffusion coefficient.

#### 3.6.2 NVT-NVE MD simulation results

**Fig. 9:**
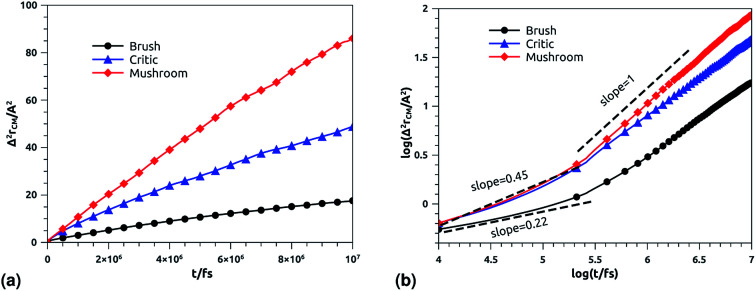
Mean square displacement of confined chitosan chains: (a) 3 dimensions MSD as a function of time, (b) log–log of MSD as a function of time.

**Fig. 10:**
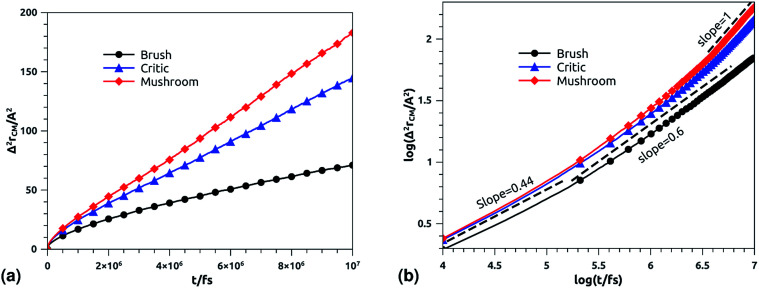
Mean square displacement of membranes: (a) 2 dimensions MSD as a function of time for DPPC molecules (the component of MSD in the *z*-direction is neglected), (b) log–log of MSD as a function of time for DPPC molecules.

**Fig. 11:**
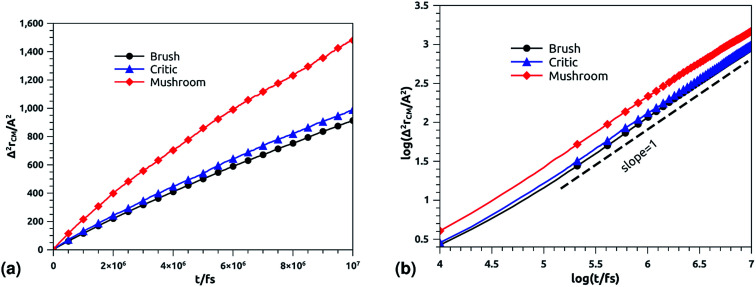
Mean square displacement of confined ions: (a) 3 dimensions MSD as a function of time, (b) log–log of MSD as a function of time.

The dynamics laws of the system components (ions, chitosan, and lipids) are identified at the three studied chitosan conformations, using an NVT-NVE MD simulation. Fig. 9, 10 and 11 show the mean-square-displacement as a function of time in the linear–linear scale and in the log–log scale, for chitosan chains, DPPC lipids, and Cl^−^ ions, respectively. At a fixed temperature *T* = 300 K. The diffusion coefficients, that are the slopes of the best linear log–log plot of the MSD as a function of time, are given in Tables 6–8. In Fig. 9b, we depict the log–log plot of MSD against time for chitosan, for three values of the grafted chitosan molar fraction correspond to the mushroom, critic and brush regimes. We first remark that after a short initial regime of subdiffusive motion (*t* = 100 ps), the chitosan dynamics reach the normal diffusion, after this time MSDs are straight lines with a slope *α* ≃ 1, but decrease as the polymer pass from the mushroom to the brush regime. We note that *α* < 0.5 for the short time, this deviation from the Rouse model is attributed to the cage effect provoked by the high density. In a recent experimental study, the diffusion coefficient *D*_c_ of the polyelectrolyte inside a gel matrix was measured using dynamic light scattering and was found to be in the order of 10^−8^ cm^2^ s^−1^.^85^ The MD results cover the experimental findings and show a dependence of the diffusion coefficients to the grafting densities of chitosan chains.

**Table:** Diffusion coefficient *D*_*α*_ of chitosan polyelectrolyte obtained by MD simulations in the NVT-NVE statistical ensemble

Regime	*t*/fs	*α*	(*D*_α_) (cm^2^ s^−*α*^)
Brush	[0,10^5.3^]	0.22	0.09 × 10^−8^
	[10^5.3^,10^7^]	0.71	2.94 × 10^−8^
Critic	[0,10^5.32^]	0.45	0.10 × 10^−8^
	[10^5.32^,10^7^]	0.80	8.12 × 10^−8^
Mushroom	[0,10^5.31^]	0.45	0.10 × 10^−7^
	[10^5.31^,10^7^]	1	1.43 × 10^−7^

**Table:** Diffusion coefficient *D*_s_ of DPPC lipids obtained by MD simulations in the NVT-NVE statistical ensemble

Regime	*t*/fs	*α*	(*D*_*α*_) (cm^2^ s^−*α*^)
Brush	[0,10^5.2^]	0.44	0.50 × 10^−7^
	[10^5.2^,10^7^]	0.6	1.77 × 10^−7^
	[0,10^5.1^]	0.44	0.62 × 10^−7^
Critic	[10^5.1^,10^6.7^]	0.6	1.25 × 10^−7^
	[10^6.7^,10^7^]	1	3.61 × 10^−7^
	[0,10^5.1^]	0.44	0.66 × 10^−7^
Mushroom	[10^5.1^,10^6.5^]	0.6	2.46 × 10^−7^
	[10^6.5^,10^7^]	1	4.57 × 10^−7^

**Table:** Diffusion coefficient *D*_*α*_ of confined ions obtained by MD simulations in the NVT-NVE statistical ensemble

Regime	*t*/fs	*α*	(*D*_s_) (cm^2^ s^−*α*^)
Brush	[0,10^7^]	1	1.52 × 10^−6^
Critic	[0,10^7^]	1	1.64 × 10^−6^
Mushroom	[0,10^7^]	1	2.47 × 10^−6^

In Fig. 10b we depict the log–log plot of MSD against time for DPPC lipids. The dynamics behavior of DPPC lipids can be affected by the presence of incorporated DPPC-chitosan lipo-polyelectrolyte with different grafting molar fraction. For a pure DPPC lipid bilayer with different molar fractions of incorporated DPPC-PEG lipo-polymer, we observed anomalous diffusion of lipids with a scaling exponent *α* < 1, depending on time. Also, we found that the calculated diffusion coefficient of lipids, at large time *t* ≥ 10^6.6^ fs, is of the order of 10^−8^–10^−7^ cm^2^ s^−1^ and decreases proportionally to the DPPC-PEG molar fraction. This dependence on the molar fraction is proved experimentally by M. L. Wagner and L. K. Tamm using fluorescence recovery after photobleaching (FRAP).^86^ In their work, the authors found that high lateral lipid diffusion is observed, in supported lipid bilayers on a polyethyleneglycol (PEG) cushion, when the molar fraction of graft PEG kept slightly below the transition to the brush regime. In this work, we found that the scaling exponent for DPPC lipid follows the same instance, *α* < 1. But, we note that *α* found for DPPC decorated with chitosan polyelectrolyte is slightly lower for comparison to that found for DPPC membranes decorated with PEG neutral polymer. This can be explained by the fact that the PEG polymer chains are more flexible compared to those of chitosan. On the first hand, the calculated diffusion coefficient of lipids, in the present study, is also found in good agreement with the previous MD simulations of DPPC membranes with incorporated neutral polymers. On the other hand, the obtained values of *D*_*α*_ cover the experimental findings.^86–88^

In Fig. 11b, we depict the log–log plot of MSD against time for ions, for three values of the grafted chitosan molar fraction correspond to the mushroom, critic and brush regimes. We first remark that the ions dynamics follow a normal diffusion, the MSDs curves are straight lines of the same diffusion exponent *α* = 1, but decrease as the polymer pass from the mushroom to the bush regime. The calculated *D*_c_ for Cl^−^ ions in the chitosan branched polyelectrolyte, confined between two adjacent liposomes, is of the order of 10^−6^ cm^2^ s^−1^, these values are in good agreement with the experimental measurements of diffusion coefficients in aqueous solutions,^89^ and are in accord with the calculated values from recently published works using all-atom MD simulations.^90^

We note that the ballistic regime is not observed for all constituents, this can be explained by the fact that the simulated system is previously equilibrated in the NPT-NPH MD simulation.

## 4 Conclusion

The aim of this study is to examine the structural and dynamic properties of DPPC membranes covered by the chitosan chains using a coarse-grained MD simulation method, with particular emphasis on the application of this system in drug delivery. Chitosan chain conformations are characterized in the NPT-NPH conditions, as a function of the grafting molar fraction *X*_p_, through calculating the length of the chitosan layer. On one hand, the obtained results are found to be consistent with the theoretical investigations, which predicts three different conformations of chitosan chains depending on *X*_p_, namely, mushroom, critic and brush regimes. On the other hand, the calculated lengths of chitosan layers are found in agreement with the available studies on neutral PEG polymer, from MD simulation and X-ray experiment. The dynamics properties have been investigated in the NVT-NVE conditions, the dynamics of the membrane slowing during the transition from the mushroom regime to the brush regime. The chitosan chains move freely following a normal diffusion and increase the dynamic flexibility of the liposomes. The ions follow a normal diffusion for all the conformation regimes independent of diffusion time. The calculated diffusion coefficients for all constituents namely, DPPC lipids, chitosan chains, and ions, are found in good agreement with the experimental ones, available in the literature. Regarding the application of chitosan covering liposomes in drug delivery, the present findings confirm the role of coated chitosan in the protection of liposomes against the adhesion phenomena expected between liposomes–liposomes and liposomes–target cells. Also, these findings provide additional information about the role of chitosan in the stabilization and the lubrication of liposomes. Thus, these results will provide a quantitative basis for designing liposomes covered with chitosan for drug delivery applications. Finally, we underline that grafting chitosan to the liposomes can also affect other properties of its membranes bilayer, for example, the melting temperature and the elastic properties.

## Conflicts of interest

There are no conflicts to declare.

## Supplementary Material
